# Paccianon the Neglected Galenic Drug for Ophthalmic Lesions

**DOI:** 10.7759/cureus.74605

**Published:** 2024-11-27

**Authors:** Gregory Tsoucalas, Efstathios T Detorakis, Andreas Manios

**Affiliations:** 1 Department of History and Medical Deontology, School of Medicine, University of Crete, Heraklion, GRC; 2 Department of Ophthalmology, University Hospital of Heraklion, Heraklion, GRC; 3 Department of Plastic Surgery, University Hospital of Heraklion, Heraklion, GRC

**Keywords:** ancient greece, collyrium, eye-salve, gaius paccius africanus, ophthalmology

## Abstract

The Greco-Roman physician Galen of Pergamon was the first to mention a drug named Paccianon. This drug was unknown in ancient Greece and most probably through the School of Alexandria entered medical literature. Oribasius and Aetius were the only two practitioners who mentioned it after Galen, administrating it in various forms, such as poultice, mixture, and collyrium, for a series of ophthalmic diseases and cutaneous lesions. Modern medicine uses some of the material used in Paccianon’s formula, proving its anticancer, antimicrobial, and anti-inflammatory properties. Paccianon constitutes one more example of the past that is significant in contemporary pharmaceutical chemistry. The drug was neglected through time, but its formula testified to the deep knowledge of the ancient Greeks in the botanical and mineral properties of various materials.

## Editorial

Paccianon (Greek: Πακκιανόν) was a therapeutic mixture used in antiquity to confront various ophthalmic infectious, inflammatory, and tumorous diseases. The survey of the ancient Hellenic literature inside the digitized database Thesaurus Linguae Graecae (TLG) unveiled only a small series of references, while the Great Lexicon of the Greek Language by Liddell and Scott does not present such an entry [1]. Galen of Pergamon (ca. 130-200 AD) was the first to name this drug in his majestic galenic pharmacology “De Compositione Medicamentorum Secundum Locos Libri X.” He supposed that Paccianon could be used to treat ulcers, demonstrating contamination (meaning infection and/or inflammation) of various tissues in the eyes, ears, and mouth. Moreover, trachoma, ecchymosis, fissures (Greek: σύκωσις, as in fig skin or incision), prominences, blisters, and burns could be confronted as well. Thus, Paccianon was used for ophthalmic diseases and cutaneous lesions, even in areas far from the eyes. The mixture was prepared with copper, peppercorns, crocus (saffron), and wine from the Aegean island of Chios, or sweet Cretan wine, mixed in a bronze pyxis (Greek: πυξίς, a small round cup-like plate) until up they thicken like a poultice. Other recipes include wine from unripe grapes, ammonia, honey from Attica, myrrh, and muddy earth made from a stone (most probably crushed, a kind of therapeutic earth) [2]. Two centuries later, Greek scholar and physician Oribasius (ca. 320-403 AD) mentioned Paccianon, adding cadmium, hematite (bloodstone), dry rose petals, Indian nard, opium, white pepper, and gum to its recipe. Allegedly, its effect could be enhanced with the use of egg yolk [3]. Both those figures of ancient Greek medicine emphasize its use in ophthalmology. Paccianon blunt pterygium reduces the erosion of the cornea which may extend to the uvea, calms inflammation, and treats trachoma, being considered a great anti-infective, pain killer, antiseptic, and styptic drug [2,3]. Byzantine writer and physician Aetius Amidenus (ca. mid-fifth to sixth century) used Paccianon as an “enstakton” (Greek: ένστακτον, instillation), a synonym of collyrium, providing an altered cluster of materials, cadmium, mismus, antimony, copper sulfate, white pepper, opium, and gum. Aetius suggested that this drug may enhance eye vision and remove eye calluses [4].

In ancient Greece, the term "collyrium" had a broader meaning, referring to any liquid medicine instilled into the eyes for the treatment of eye diseases, as well as paste, ointment, and fine clay. Therefore, all forms of Paccianon throughout the ages could have similarities in texture. Oribasius used the pompous adjective “Asclepiadeum” (Greek: Ασκληπιάδειον) to signify that this is a medical remedy, or perhaps even more, to accent the drug to divine medication status, associating its origin from god Asclepius, the patron of medicine in ancient Greece, and presenting it as a panacea for a limitless use [1]. Galen used the adjective “crocodes” (Greek: κροκώδες), to annotate its texture and its substance, which is full of crocus [2]. Crocus had a significant position in traditional medicine and herbal remedies since the era of the Minoan civilization [5]. Physician and botanist Pedanius Dioscorides (ca. 40-90 AD) in his masterpiece “De Materia Medica,” proposed crocus against rheumatism of the eyes, that is against morbid fluids secreted from or circulating within the eye [6], as Oribasius did [3]. Modern phytochemistry proved crocus to have significant potential with anticancer, antidiabetic, antioxidant, antimicrobial, anti-inflammatory, antinociceptive, anticonvulsant, antidepressant, learning and memory enhancement, cardiovascular, and antihypertensive properties [7]. Researchers studied petal and stigma extracts from crocus plants (*Crocus sativus *L.), demonstrating antibacterial capacity against *Bacillus subtilis*, *Escherichia coli*, *Listeria monocytogenes*, *Proteus vulgaris*, *Pseudomonas aeruginosa*, *Salmonella enterica*, and *Staphylococcus aureus*, suggesting that crocus can be used as a natural agent in the pharmaceutical industry [8]. The formula of Pacciano except from herbal material included a series of mineral ones. Contemporary clashes against bacteria boosted antimicrobial resistance, with a continuous decline in the effectiveness of a series of conventional antimicrobial therapies. A renewed interest in exploring the potential of metal-based antimicrobial compounds is now in vogue. Copper itself was widely used too [6], while modern chemistry proved its anti-inflammatory and antitumorous capabilities [9]. Furthermore, antimony compounds such as SbPh4ACO have been successfully tested in strains of *Pseudomonas aeruginosa*,* Escherichia coli*, and *Staphylococcus aureus*, and may be used as potentiators for other antibiotics against both Gram-positive and Gram-negative bacterial pathogens [10]. Galen noted that Erasistratus of Ceos (ca. 304-250 BC) had knowledge of the drugs and used them to treat ophthalmic diseases containing crocus and mineral compounds, as he and Erasistratus had been trained in Alexandria, being acquainted with such remedies due to the high prevalence of ophthalmic diseases among the Egyptians [2,11]. It seems that most compounds of Pacciano drug have been proven to present a healing action. Wine, antiseptic; psychotropic; honey, skin nutrient and barrier; anti-itching; myrrh, antiseptic, painkiller, and anticallous; cadmium (or cadmia), antiseptic; "cerotis" (waxer, skin barrier); hematite, blood nutrient and antitumorous; earth, antiseptic, antimicrobial, nutrient, skin barrier, and anti-inflammatory; and all other herbs and minerals, which were found to be therapeutic after long observations by the ancient Greek medico-philosophers, rhizotomoi (root cutters, botanists), and physicians [6]. The use of such mixtures, and especially the technique of egg yolk refinement, was adopted centuries later by the physicians and surgeons of Western Europe, as in the case of the French barber-surgeon Ambroise Paré (ca. 1510-1590 AD). Paré widely used mixtures of herbs and minerals, boosted with egg yolk, for their antiseptic and styptic character [12]. The eye salve technique for treating eye diseases dates back to the era of the first pre-Socratic philosophers and was used by the Hippocratics in ophthalmology. Although many formulas were mentioned, none was named Paccianon, nor did the term exist, that is, until the time of Galen [1]. This eye salve, Paccianon, could have been named after Gaius Paccius Africanus (ca. first century AD), a Roman official, proconsul, and governor of the province of Africa (bordering Egypt and the Medical School of Alexandria) in the Roman Empire [11]. Paccius may have suffered from an eye ailment and been successfully treated with such a mixture, making it famous in the region and leading it to enter Galenic pharmacology [2,11]. Other suggestions regarding Paccianon’s nomination may also include as follows: (i) Paccius Antiochus, the physician (first century AD), a student of Philonides of Catana, became famous for his medicinal preparations and a panacea he composed for Emperor Tiberius [2,13,14]; and (ii) Paccius, the friend of the philosopher Atticus (ca. second century AD), to whom Plutarch (ca. 45-120 AD) dedicated some treatises, such as the one 'On Joy' (Greek: Περί Ευθυμίας) [15].

The Paccianon mixture, which may have entered medicine through the Alexandrian School, was a complex medicinal formula - whether as a salve, poultice, or liquid depending on its use - a pioneering achievement in ancient Greek medicine. It was used by Galen, Oribasius, and Aetius for the treatment of ailments, primarily of the eyes and skin. Its composition and alternatives demonstrated advanced knowledge in botany and empirical "chemistry," as the formulation combined materials with different healing properties for targeted therapies.
